# TLR4 Asp299Gly and Thr399Ile Polymorphisms: No Impact on Human Immune Responsiveness to LPS or Respiratory Syncytial Virus

**DOI:** 10.1371/journal.pone.0012087

**Published:** 2010-08-10

**Authors:** Renée N. Douville, Yuriy Lissitsyn, Aaron F. Hirschfeld, Allan B. Becker, Anita L. Kozyrskyj, Joel Liem, Nathalie Bastien, Yan Li, Rachel E. Victor, Mehtab Sekhon, Stuart E. Turvey, Kent T. HayGlass

**Affiliations:** 1 CIHR National Training Program in Allergy and Asthma Research, Department of Immunology, University of Manitoba, Winnipeg, Canada; 2 Department of Medical Microbiology, University of Manitoba, Winnipeg, Canada; 3 Department of Community Health Sciences, University of Manitoba, Winnipeg, Canada; 4 Department of Pediatrics/Child Health, University of Manitoba, Winnipeg, Canada; 5 Canadian Science Centre for Human and Animal Health, Winnipeg, Canada; 6 Department of Pediatrics, University of British Columbia, BC Children's Hospital and Child and Family Research Institute, Vancouver, Canada; Louisiana State University, United States of America

## Abstract

**Background:**

A broad variety of natural environmental stimuli, genotypic influences and timing all contribute to expression of protective versus maladaptive immune responses and the resulting clinical outcomes in humans. The role of commonly co-segregating Toll-like receptor 4 (*TLR4*) non-synonymous single nucleotide polymorphisms Asp299Gly and Thr399Ile in this process remains highly controversial. Moreover, what differential impact these polymorphisms might have in at risk populations with respiratory dysfunction, such as current asthma or a history of infantile bronchiolitis, has never been examined. Here we determine the importance of these polymorphisms in modulating LPS and respiratory syncytial virus (RSV) - driven cytokine responses. We focus on both healthy children and those with clinically relevant respiratory dysfunction.

**Methodology:**

To elucidate the impact of *TLR4* Asp299Gly and Thr399Ile on cytokine production, we assessed multiple immune parameters in over 200 pediatric subjects aged 7–9. Genotyping was followed by quantification of pro- and anti-inflammatory cytokine responses by fresh peripheral blood mononuclear cells upon acute exposure to LPS or RSV.

**Principal Findings:**

In contrast to early reports, neither SNP influenced immune responses evoked by LPS exposure or RSV infection, as measured by the intermediate phenotype of pro- and anti-inflammatory cytokine responses to these ubiquitous agents. There is no evidence of altered sensitivity in populations with “at risk” clinical phenotypes.

**Conclusions/Significance:**

Genomic medicine seeks to inform clinical practice. Determination of the *TLR4* Asp299Gly/Thr399Ile haplotype is of no clinical benefit in predicting the nature or intensity of cytokine production in children whether currently healthy or among specific at-risk groups characterized by prior infantile broncholitis or current asthma.

## Introduction

Genomic medicine seeks to inform clinical practice. It is widely anticipated that identifying specific components of an individual's genome will help determine the optimal approach to health care; for example, by facilitating the prediction and prevention of diseases such as asthma [1], or by identifying optimal pharmacologic agents for therapy [2], [3]. One early example, the study of susceptibility to respiratory syncytial virus (RSV), led to much attention focused on genes that encode proteins of the innate immune system. Since innate immunity forms the first line of defense against infection, and its activation shapes the ensuing protective or maladaptive antigen-specific immune responses that develop, it is hypothesized that variation in the genes encoding proteins of the innate immune system will influence human susceptibility [4]–[6].

The role of Toll-like receptor 4 (TLR4) polymorphisms in influencing clinical and immunologic responses to environmental stimuli is an area of much continuing and conflicting attention. TLR4 is specifically involved in generating immune responses against a diverse panel of agonists [7], including controversially, RSV. Kurt-Jones et al initially reported that TLR4 is key in initiating innate immune responses of monocytes to the fusion (F) protein of RSV [8]. These early human data were supported by experimental animal studies in which RSV was found to persist longer in the lungs of TLR4-deficient mice compared to TLR4-expressing controls [9]. At the same time, the validity of these findings and their broader impact on immune capacity in diverse human populations has remained controversial, as others, in both animals and human populations have not replicated these results [10], [11].

The human *TLR4* gene harbours two commonly co-segregating single nucleotide polymorphisms (SNP)—Asp299Gly and Thr399Ile—that alter the amino acid sequence of the TLR4 protein. Experimental systems indicate that the 299Gly SNP is associated with hyporesponsiveness to TLR4 ligands such as LPS *in vivo* and *in vitro*, and that it acts in a dominant fashion with respect to the more common Asp299 allele [12], [13]. Currently there is little consensus on how such genetic factors controlling the innate immune system influence the outcomes of endotoxin exposure or RSV infection in otherwise healthy children. By way of example, one study demonstrated that children requiring admission to hospital for RSV infection carried the *TLR4* 299Gly allele at a higher frequency than children with mild disease not requiring hospitalization [14], however, subsequent independent work by our group did not identify such a positive association [15], and another group observed a weak association in the opposite direction where the more common Asp299 allele was overrepresented in children with severe RSV infection [16]. Most recently, heterozygosity of Asp299Gly and Thr388Ile was found to be highly associated with symptomatic RSV disease in high-risk infants [17]. However, the confounding impact of known clinical risk factors for severe RSV infection in this cohort, including prematurity and bronchopulmonary dysplasia, made generalization of these data challenging.

LPS and RSV are ubiquitous in the environment. Pediatric populations may experience substantially higher exposure to both agents and exhibit a higher burden of disease than adults. Given the morbidity associated with RSV bronchiolitis and the observation that RSV infection increases the risk of subsequent wheezing and asthma, we undertook a rigorous examination of the functional impact of the *TLR4* Asp299Gly and Thr399Ile polymorphisms on the human immune response to both LPS exposure and, independently, to RSV infection.

The most significant challenges associated with prior studies of this question include (i) use of cell lines, reliance on artificial experimental systems in which cell lines are transfected with *TLR4* variants, or use of inbred animal models, the results of which are often directly extrapolated to highly diverse human populations, (ii) use of human cells ex vivo as previously frozen samples or following pre-conditioning regimens with addition of cytokines (ie IFNγ) to culture, both of which have demonstrated potential to skew the resulting immune responses, (iii) small sample sizes (frequently less than 100), and (iv) use of different, or undefined, variants of RSV for re-stimulation. Importantly, the majority of studies attempt to link SNPs or haplotypes directly to clinical outcomes, without analysis of the intermediate phenotype of immune function. Thus, notwithstanding extensive evidence of the impact of gene by environment by time interactions on a wide variety of immune and clinical outcomes [18]–[20], many studies to date, due to their experimental design, cannot evaluate the impact of these factors on immune capacity and LPS or RSV specific responses.

As an alternative approach to better elucidate the immunological impact of the *TLR4* Asp299Gly and Thr399Ile polymorphisms on putatively distinct cytokine production in human populations that interact with a broad diversity of natural environmental stimuli throughout childhood, we measured multiple immune parameters in over 200 pediatric subjects aged 7–9 years following acute *in vitro* re-exposure to LPS or RSV. The results demonstrate that while some reports link *TLR4* Asp299Gly polymorphism with clinical outcomes of RSV infection, the haplotype comprised of Asp299Gly and Thr399Ile does not detectably impact the immune response to RSV, as measured by the intermediate phenotype of RSV-stimulated cytokine responses nor LPS-driven cytokine production. This conclusion applies equally to healthy children and those with “at risk clinical phenotype” characterized by current asthma or a history of severe bronchiolitis in infancy.

## Materials and Methods

### Recruitment of child participants and ethics statement

Study approval was obtained from the University of Manitoba Faculty Committee on Use of Human Subjects in Research. This 1995 birth cohort (SAGE, Study of Allergy Genes and Environment) was created from the Manitoba Canada universal provincial health care registry [20]. Written informed consent was obtained for 723 children (of whom 207 were randomly selected for detailed analysis) within a general population survey case-control cohort. Consented children and parents came to the Pediatric Allergy Clinic in Winnipeg or mobile Pediatric Allergy clinic in their community for the detailed assessment used to characterize the individuals studied in this publication.

In prior studies, 100% of this age group was seropositive for prior RSV infection, reinforcing the ubiquity of these infections in the general population. These 7–9 year old children had no evidence of current upper or lower respiratory tract infection within one month of recruitment based on histories from child and parent (no “colds”) and no current evidence of a URI on physical examination.

### Clinical assessment of asthma in children

Pediatric allergist assessment was used as the gold standard for diagnosis of current asthma (ie. within the last 12 months). Diagnosis was based on history and physical assessment (blinded to skin prick tests and PC_20_), according to the Canadian Asthma Consensus Guidelines [21]. This incorporated a standardized history and physical sheet, questions regarding cough with/without colds, wheeze with/without colds, shortness of breath with activities, colds lasting >2 weeks, response to current medications and the presence of other allergic conditions, family history of asthma or personal history of prior/present eczema, hospital, emergency department and medical visits for breathing difficulty in the past year, and physical examination for allergic facies, chest findings and evidence of atopic dermatitis.

### Epidemiologic parameters

Bronchiolitis was defined as an ICD-9 (International Classification of Diseases) diagnosis of “acute bronchitis and bronchiolitis” (466 code) between birth and age 2, which was identified using the Manitoba Center for Health Policy databases.

### PBMC isolation

Blood was collected in tubes containing EDTA, peripheral blood mononuclear cells (PBMC) were isolated by density centrifugation, counted (>95% viability by trypan blue exclusion), and used immediately for short-term primary culture in the absence of exogenously added cytokines.

### Generation of viruses for cell culture

RSV strain Long was cultured on Hep-2 cells at 37°C in Eagle's MEM supplemented with penicillin (100 U/ml), streptomycin (100 µg/ml), 30 µg/ml L-glutamine (Life Technologies) and 1% fetal calf sera (Sigma). RSV was titrated by the quantal assay TCID_50_ performed in 96-well microtiter plates using tenfold dilutions.

### Primary cell culture

Freshly isolated PBMC were suspended in complete medium (RPMI 1640 with 10% heat-inactivated fetal calf serum, 1% penicillin/streptomycin/fungizone, 0.3 mg/ml L-glutamine and 0.1% 2-mercaptoethanol) with 2.5×10^6^ cells/ml in 200 µl in 96 well U bottom plates. Duplicate cultures were stimulated with LPS (BioXtra, L4391) from Escherichia coli serotype 00011:B4 at 0.05, 0.5 and 5.0 ng/ml from Sigma (St. Louis, MO) or RSV strain Long (10^4.9^ TCID_50_/ml). LPS purity was validated by i) its inability to activate other human TLRs expressed in transfected HEK 293 cells, and ii) the finding that pretreatment with TLR4 blocking antibodies (10 ug/ml anti-CD284/TLR4, Ebioscience, San Diego, CA), but not IgG2a isotype control antibodies (Ebioscience), blocks the capacity of LPS to stimulate cytokine production demonstrate its specificity for TLR4 (**Figure S1**). Based on data obtained in preliminary time course experiments (data not shown), culture supernatants were harvested following 1 or 6 days culture, the respective time of peak LPS and RSV driven responses for the cytokines evaluated. The optimal concentration of LPS stimulation used to evaluate IL-10 and CCL2 responses was 0.05 ng/ml, with 0.5 ng/ml for IL-1β and TNFα responses. While the intensity of responses evoked was weaker in most individuals when sub-optimal concentrations were used, the conclusions from the data were identical (data not shown).

### Human cytokine ELISA

As a safety precaution against infection, culture supernatants were UV irradiated for one hour to inactivate residual virus prior to ELISA analysis. Experiments (data not shown) demonstrated that this had no impact on the sensitivity or precision of the assays used to evaluate cytokine concentrations. Anti-cytokine capture and biotinylated detection antibodies were purchased from BD-Pharmingen (Mississauga, ON, Canada), Endogen (Woburn, MA, U.S.A.), Biolegend (San Diego, CA, U.S.A.) or R&D Systems (Minneapolis, MN, U.S.A.) and recombinant cytokine standards from BD Pharmingen, Endogen or Peprotech (Rocky Hill, NJ, U.S.A.). They were used as previously described [22], [23]. PBMC supernatants from a minimum of duplicate cultures were assayed. Each sample was then evaluated in at least two assays, with the concentration in each calculated from a minimum of three points falling on the linear portion of titration curves calibrated against recombinant cytokine standards serially diluted on each plate. Standard errors typically ranged from 3–10%. All cytokine standards and antigenic stimuli (LPS and RSV) were each derived from single lot preparations. Because a large number of different immune-assays needed to be performed on culture supernatants derived from the limited pediatric blood volumes drawn, some samples were consumed before all analyses could be completed. Thus, some variation exists in the “n” available to assess different cytokines in both the asthmatic/bronchiolitis groups and control populations based on availability of materials. No data were deliberately excluded.

### *TLR4* Asp299Gly and Thr399Ile Genotyping

The *TLR4* allelic variants Asp299Gly (refSNP ID: rs4986790) and Thr399Ile (refSNP ID: rs4986791) were genotyped by quantitative PCR assay as previously described [24]. Briefly, genomic DNA was amplified using a TaqMan® Pre-Designed SNP Genotyping Assay (Asp299Gly #C_11722238_20, Thr399Ile #C_11722237_20, Applied Biosystems) which contains primers and probes specific for each allelic variant of the SNP in question. The genotyping assay was performed using an Applied Biosystems 7300 Real Time PCR System.

### Statistical Analysis

Associations between Ag-driven responses by subjects were compared using 2-tailed Mann-Whitney tests (nonparametric data). All statistics were performed using GraphPad Prism version 3.02 for Windows (GraphPad Software, San Diego, USA).

## Results

### Asp299Gly and The399Ile *TLR4* Genotyping

We genotyped 207 7–9 year old children from the SAGE cohort (Table 1). *TLR4* Asp299Gly and Thr399Ile SNPs were in Hardy-Weinberg equilibrium, with the common homozygote genotypes co-segregating in 202 of 207 individuals (97.5%). This cosegregation is consistent with data demonstrating that these SNPs are located on the same allele in European populations [25]. Rather than treating each polymorphism as an independent variable, it is most helpful to consider the *TLR4* haplotypes formed by the Asp299Gly and Thr399Ile polymorphisms, because different haplotype structures are observed in different human populations [26]. Our cohort is essentially comprised of two major haplotypes: Asp/Asp+Thr/Thr (87.0%) and Asp/Gly+Thr/Ile (10.6%) (See Table 1). Consequently, to optimize power, all subsequent analysis was limited to comparing the homozygous (Asp/Asp+Thr/Thr) and heterozygous (Asp/Gly+Thr/Ile) *TLR4* haplotypes.

**Table 1:** TLR4 SNP genotypes, allele frequencies and haplotypes.

LR4 SNP frequencies						
Asp299Gly				Thr399Ile		
	*n*	%			*n*	%
**Genotype**				**Genotype**		
Asp/Asp	183	88.4		Thr/Thr	182	87.9
Asp/Gly	24	11.6		Thr/Ile	25	12.1
Gly/Gly	0	0.0		Ile/Ile	0	0.0
*Total*	207	100.0		*Total*	207	100.0
**Allele**				**Allele**		
Asp	390	94.2		Thr	389	94.0
Gly	24	5.8		Ile	25	6.0
*Total*	414	100.0		*Total*	414	100.0

TLR4 SNP haplotype frequencies						
Asp299Gly	Thr399Ile	*n*	%			
Asp/Asp	Thr/Thr	180	87.0			
Asp/Asp	Thr/Ile	3	1.5			
Asp/Gly	Thr/Thr	2	0.9			
Asp/Gly	Thr/Ile	22	10.6			
*Total*		207	100.0			

### Common TLR4 haplotypes do not influence LPS-stimulated cytokine production

**Figure 1:**
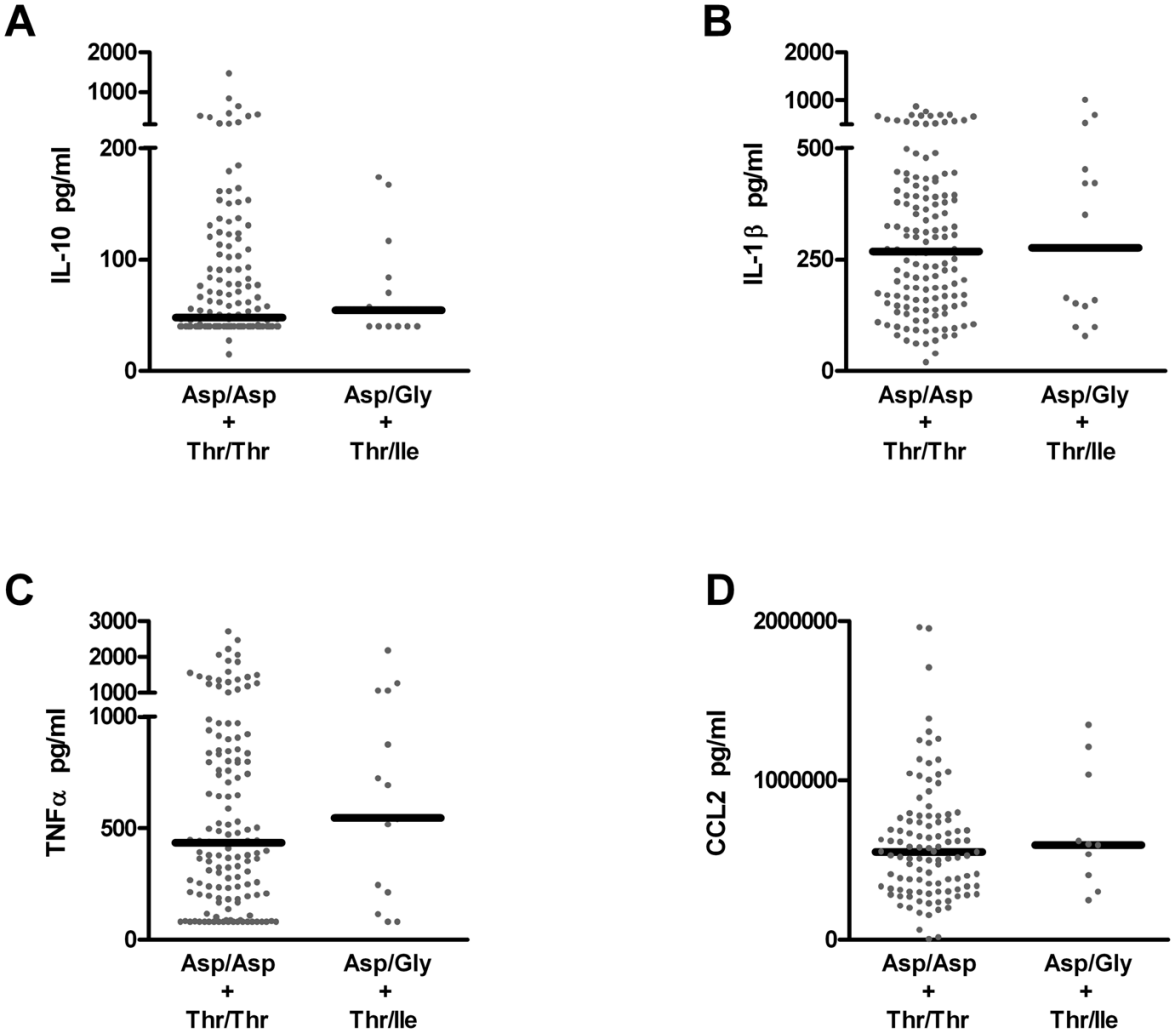
Indistinguishable LPS-driven cytokine responses are evoked in the cells of children with the homozygous (Asp/Asp+Thr/Thr) vs heterozygous (Asp/Gly+Thr/Ile) *TLR4* haplotypes. IL-10 (**A**), IL-1β (**B**), TNFα (**C**) and CCL2 (**D**) responses were quantified in supernatants from 1 day primary PBMC cultures, each from an individual child (•). Black bars represent median responses.

The impact of the *TLR4* Asp299Gly and Thr399Ile polymorphisms on LPS responsiveness remains highly controversial [12], [13], [15], [26]–[31]. Many of these studies are quite limited in size. Given the multiple environmental and genetic factors that influence and can confound assessment of innate and adaptive immune function, large sample sizes with analysis of fresh primary cells directly *ex vivo* are required. Using acute assays of primary PBMC cell cultures from 202 healthy children, we found that LPS stimulated secretion of four characteristic TLR4-driven pro- and anti-inflammatory cytokines, IL-10, IL-1β, TNFα and CCL2, was indistinguishable between subjects with the common homozygous haplotype (Asp/Asp+Thr/Thr) and those with the heterozygous haplotype (Asp/Gly+Thr/Ile) (**Figure 1**). With multiple stimulation conditions in culture (ie. different LPS concentrations, virus stimulation) and subsequent quantification of multiple cytokines in culture supernatants from these pediatric populations, not all individuals were assayed for all outcomes. In cases where cell numbers were limiting, priority was given to RSV stimulation. The use of ‘maximal’ (5 ng/ml), intermediate (0.5 ng/ml) and ‘low” (0.05 ng/ml) LPS concentrations influences the intensity of cytokine secretion in individuals; however, at none of the other concentrations studied were differences or trends seen between the two variants of the *TLR4* Asp299Gly polymorphism across this pediatric population (data not shown).

### TLR4 haplotype has no effect on LPS-driven cytokine responses in pediatric populations stratified by asthmatic status or prior bronchiolitis

**Figure 2:**
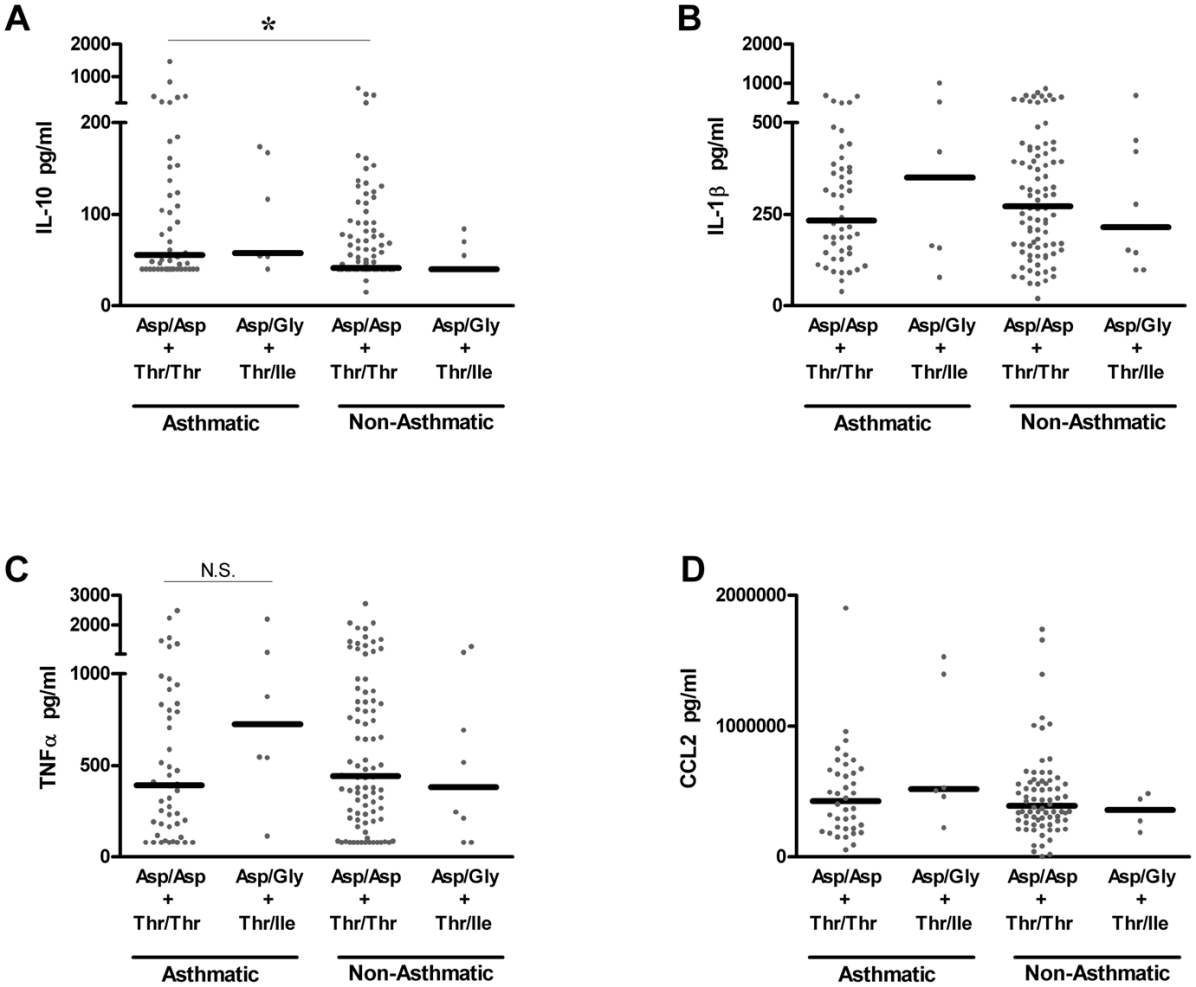
Similar LPS-driven cytokine responses are expressed by children with differing *TLR4* haplotypes, independent of current asthma. IL-10 (**A**), IL-1β (**B**), TNFα (**C**) and CCL2 (**D**) were assessed as detailed above. * P<0.05, with all other comparisons P>0.05.

**Figure 3:**
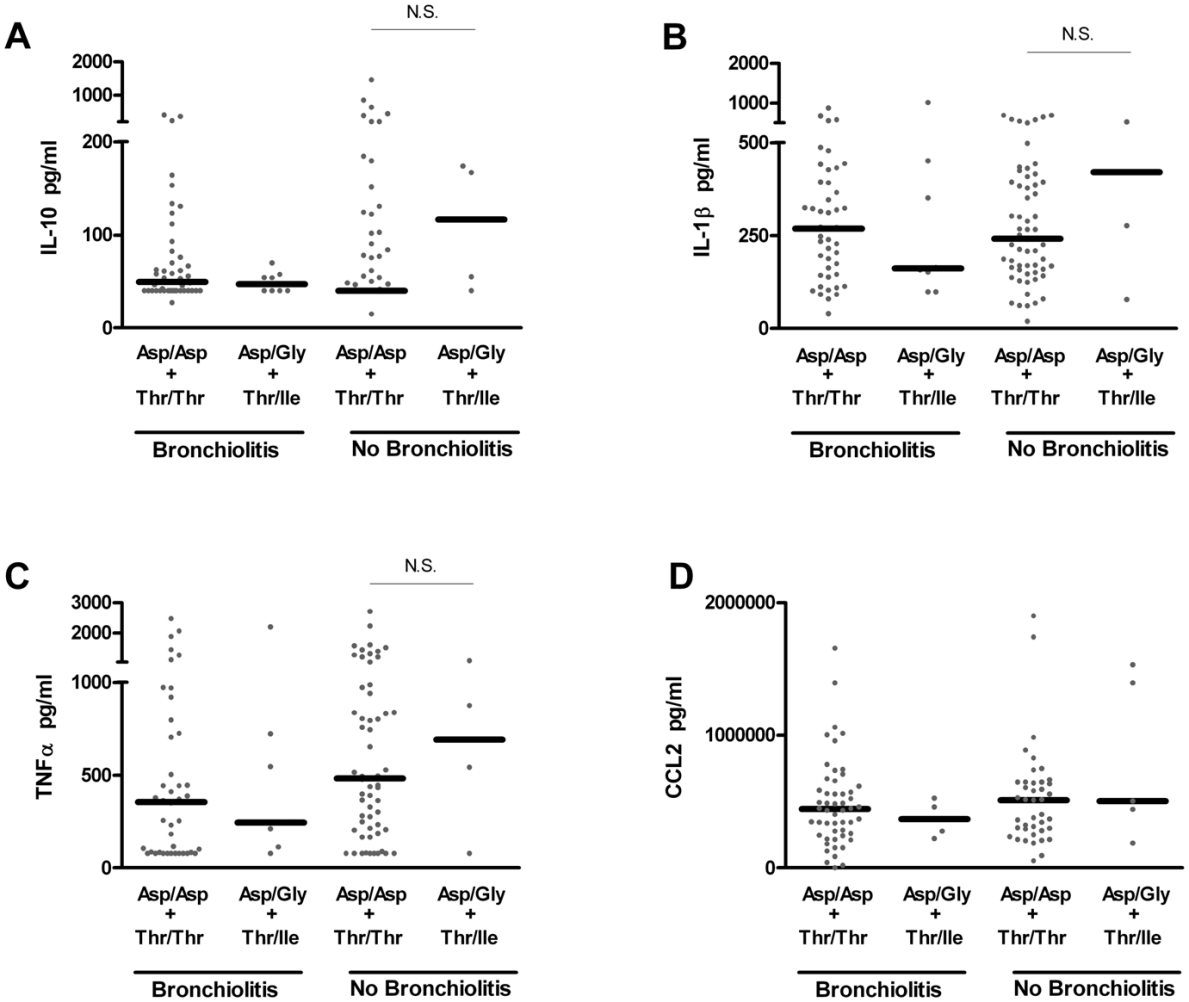
Similar LPS-driven cytokine responses exhibited by children with the homozygous (Asp/Asp+Thr/Thr) and heterozygous (Asp/Gly+Thr/Ile) *TLR4* haplotypes with or without prior bronchiolitis. All comparisons yield P>0.05.

Many functionally important genetic associations are better revealed in at-risk groups than in general population studies. Given that some prior studies, often with an *n* of 20–100, demonstrated an association between *TLR4* polymorphisms and LPS hyporesponsiveness or clinical outcomes of airway dysfunction such as asthma and bronchiolitis [14], [26], [31], [32], we conducted subanalyses of genotype and intermediate phenotype based on stratification by (i) current asthma (**Figure 2**) or a prior clinical history of bronchiolitis in the first two years of life (**Figure 3**).

Among children carrying the homozygous haplotype (Asp/Asp+Thr/Thr), asthmatics produce higher levels of IL-10 (p<0.03) in response to stimulation with LPS relative to non-asthmatics (**Figure 2**). This supports the body of literature indicating differences between asthmatics and non-asthmatics in their innate immune capacity. However, stratification of children based on current asthma or prior bronchiolitis or the absence of either condition, consistently demonstrates an indistinguishable capacity to express LPS-driven cytokine production between the two *TLR4* haplotypes.

### Similar RSV-driven cytokine responses in children with homozygous or heterozygous TLR4 haplotypes

**Figure 4:**
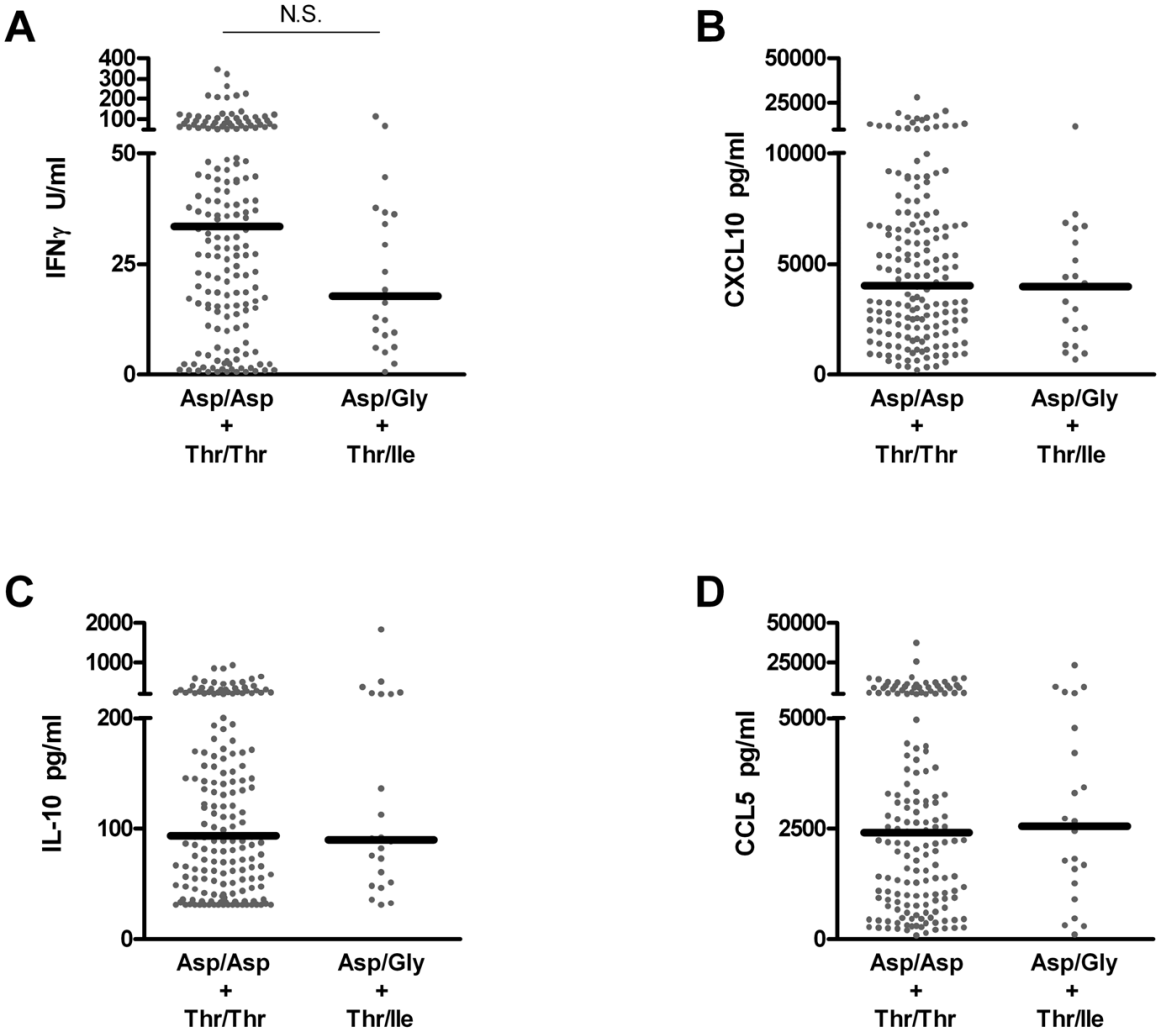
Indistinguishable responses are elicited by respiratory syncytial virus re-exposure in children with differing *TLR4* haplotypes. RSV-dependent responses were measured in culture supernatants following six days primary culture. IFNγ (**A**), CXCL10 (**B**), IL-10 (**C**) and CCL5 (**D**) were measured. All comparisons yield P>0.05.

Results obtained from fresh primary PBMC culture derived from the 207 children examined in this cohort demonstrate that the intensity or nature of anti-viral immune responses driven by RSV infection are not influenced by the *TLR4* haplotype (**Figure 4**). RSV driven cytokine production is indistinguishable between these groups, regardless of haplotype.

### TLR4 haplotype has no effect on RSV-driven cytokine responses in children stratified by asthmatic status or prior bronchiolitis

**Figure 5:**
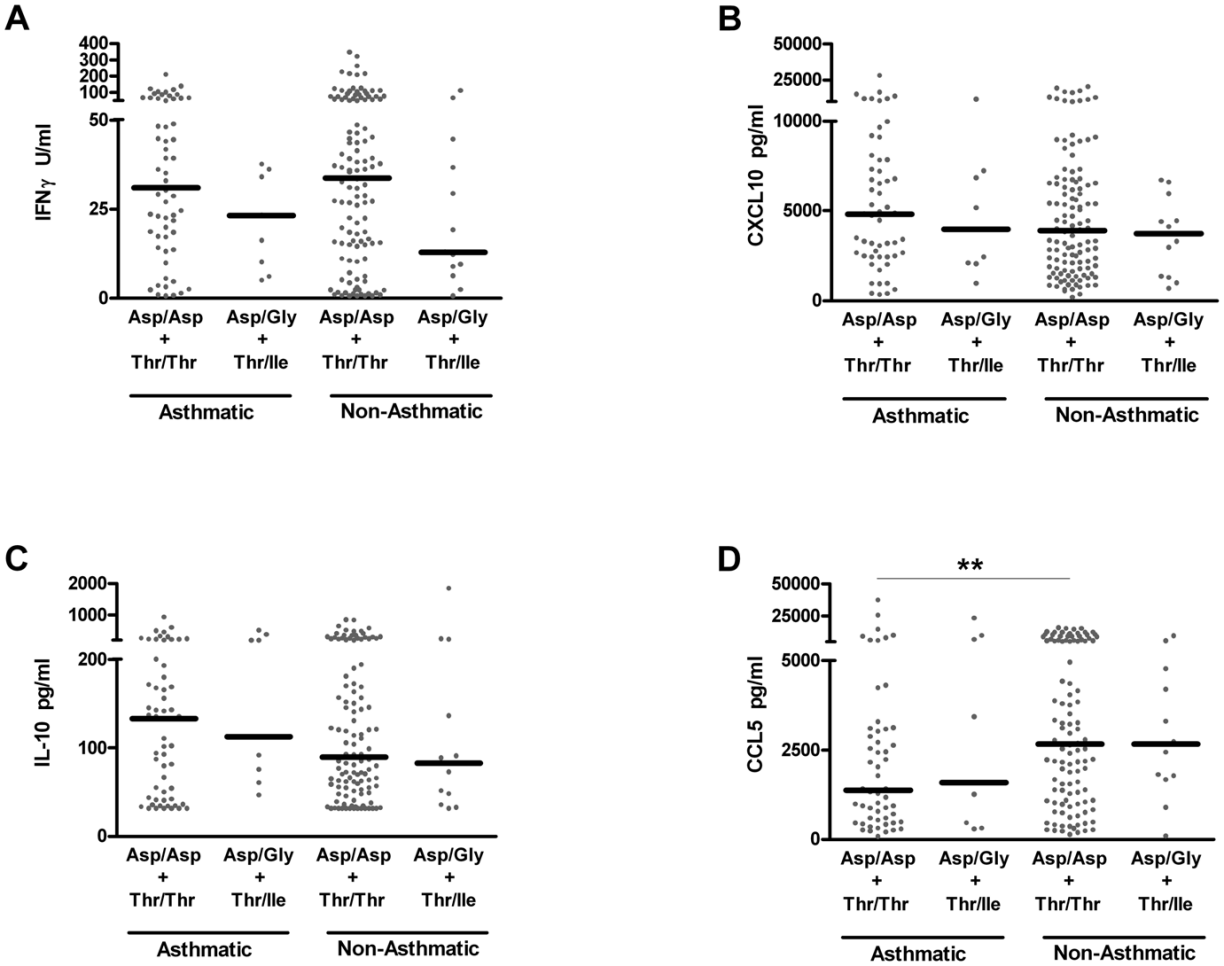
RSV stimulated cytokine responses in children or without current asthma are not influenced by *TLR4* haplotypes. IFNγ (**A**), CXCL10 (**B**), IL-10 (**C**) and CCL5 (**D**) were measured by ELISA in supernatants from 6 day PBMC cultures. Black bars represent median responses, each from an individual child (•). ** P<0.01, with all other comparisons P>0.05.

More targeted analysis, via stratification of children based on asthma or prior bronchiolitis in infancy, also reveals indistinguishable responses in virus-stimulated cytokine production between individuals with the homozygous *TLR4* haplotype (Asp/Asp+Thr/Thr) or the heterozygous haplotype (Asp/Gly+Thr/Ile) (**Figures 5** **and** **6**). These data argue against a functional impact on cytokine production between these haplotypes. In contrast, the identification of differential CCL5 production between asthmatic and non-asthmatic children in response to RSV stimulation (**fure 5D**, p<0.01), underlines the capacity and sensitivity of this method at distinguishing differences in immune capacity between these populations [22], [23].

**Figure 6:**
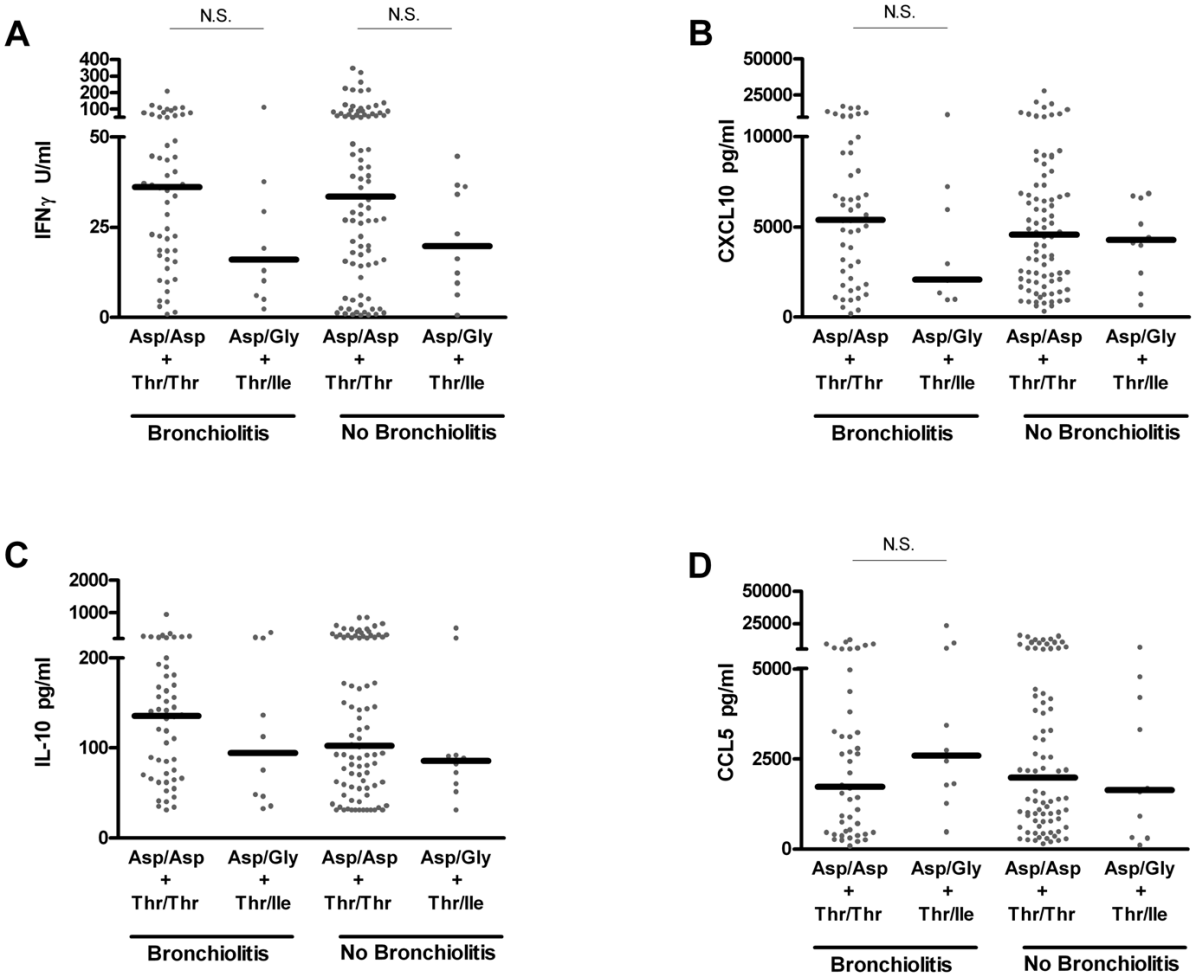
Prior bronchiolitis/bronchitis does not alter current RSV-driven cytokine responses in cells from children with the homozygous (Asp/Asp+Thr/Thr) and heterozygous (Asp/Gly+Thr/Ile) *TLR4* haplotypes. IFNγ (**A**), CXCL10 (**B**), IL-10 (**C**) and CCL5 (**D**) were measured by ELISA in supernatants from 6 day PBMC cultures. Black bars represent median responses, each from an individual child (•). All comparisons yield P>0.05.

## Discussion

The role of the haplotype comprised of *TLR4* Asp299Gly and Thr399Ile polymorphisms in shaping immune activation and subsequent clinical outcomes in humans remains highly controversial. Much of this controversy may stem from the diversity of experimental systems employed. Here, using freshly derived *in vitro* stimulated PBMC in short term culture, we determined whether children with the *TLR4* heterozygous haplotype differ from the majority of the population, which carries the homozygous haplotype, in their capacity to mount LPS or RSV-driven immunoregulatory cytokine responses. This cohort of >200 individuals demonstrates that both LPS and RSV-driven responses are indistinguishable in both groups, regardless of haplotype. Further stratification of the cohort based on clinical phenotype (current allergic asthmatic status) also revealed similar LPS- and RSV-driven responses among individuals of the two genotypes. A third independent approach, with cohort stratification based on a history of moderate to severe bronchiolitis/bronchitis during early infancy, also revealed that indistinguishable cytokine responses are elicited in these populations. Thus, the *TLR4* Asp299Gly + Thr399Ile heterozygous haplotype has no detectable effect on the capacity of randomly selected 7–9 year old children to mount either RSV-stimulated or LPS-driven responses regardless of whether the children exhibited a clinical phenotype of current asthma or those with a history of infantile bronchiolitis.

The diversity of findings evident in the literature from examination of the putative effects of the *TLR4* Asp299Gly polymorphism on LPS responsiveness is likely affected by the disparity in the cosegregation frequency of the Asp299Gly and Thr399Ile alleles in various study populations. In the only large study completed to date other than our own, (n = 245 African subjects) LPS-induced cytokine production by PBMC was not influenced by *TLR4* Asp299Gly genotype [29]. Our data extend these findings when PBMC (mainly from white children) are stimulated with LPS at both lower and intermediate concentrations that may reflect environmental conditions better than the prior widespread use of high concentrations of TLR ligands.

Interestingly, among the 64 statistical comparisons made between the two haplotypes in this study which showed no statistically significant differences in production of a variety of cytokines, a single exception was observed — that amongst children with prior bronchiolitis. Children with prior bronchiolitis in infancy who were carriers of the common homozygous *TLR4* haplotype (Asp/Asp+Thr/Thr) produced marginally higher levels of IL-10 than did carriers of the heterozygous haplotype (Asp/Gly+Thr/Ile) if their cells were stimulated at the higher concentration (0.5 ng/ml) of LPS (p<0.04) (**Figure S2**). None of the other cytokines tested in this population differed. While this borderline statistical significance may be a consequence of multiple comparisons, it does raise the possibility that under conditions of increased LPS exposure, *TLR4* haplotype may influence LPS-driven IL-10 production in select children who exhibited severe pathological symptoms during RSV infection in infancy. Further experiments specifically addressing this hypothesis, in a larger cohort specifically constructed for this purpose, would be required to discriminate between these possibilities.

Despite extensive evidence of the impact of gene by environment by time interactions on a wide variety of immune and clinical outcomes, studies to date, due to their experimental design, were unable to evaluate the impact of the *TLR4* Asp299Gly polymorphism on RSV specific immune responses from individuals with a prior history of bronchiolitis or airway dysfunction. Here, we used the intermediate phenotype of RSV-specific cytokine responses to attempt to bridge the gap between genotype and clinical phenotype. In contrast to the recent study by Tulic et al., which found associations between RSV-driven cytokine production and the Asp299Gly (or Thr399Ile) genotype [31], we i) examined a substantially greater number of individuals (207 versus 24), ii) chose to use fresh PBMC in the absence of pre-activation with IFNγ, iii) stimulated PBMC with a prototypic strain of RSV and iv) performed subanalysis in clinically different patient populations. We show that individuals with the Asp/Gly genotype demonstrate RSV-driven cytokine responses indistinguishable from those of individuals carrying the Asp/Asp genotype. Moreover, while addition of anti-TLR4 to LPS-stimulated cultures reduced cytokine production to background levels, inclusion of this blocking antibody had no impact on the intensity of RSV-stimulated cytokine responses (data not shown). Collectively, these data strongly argue that neither TLR4 nor these functional variants directly impact LPS stimulated or RSV-driven cytokine production in humans. However, it can never be excluded that other readouts not examined here might indicate differences.

In summary, multiple pattern recognition receptors including various TLR and RLR continue to be linked to resistance to and resolution of RSV infection in humans and animal models [32]–[37]. While some reports have linked *TLR4* Asp299Gly polymorphism with clinical outcomes of RSV infection, our data show that the haplotype comprised of Asp299Gly and Thr399Ile does not detectably impact the human immune response to RSV, as measured by the intermediate phenotype of RSV-stimulated cytokine responses or that of a well validated TLR4 ligand, LPS. In this study we did not try to directly link TLR4 SNPs to clinical outcome, but rather stratified by clinical phenotype (asthma or bronchiolitis) to see if differences might be enriched in these groups. Cytokine production from PBMC in a ∼200 member cohort, or in subcohorts characterized by current asthma or a history of severe bronchiolitis in infancy consistently yield indistinguishable pro- and anti-inflammatory cytokine production. As both LPS and RSV can also interact with alternate pattern recognition receptors [32], [38], new possibilities for genomic targets in the prediction and prevention of harmful diseases such as RSV infection and asthma await investigation.

## Supporting Information

Figure S1 Anti-TLR4 blocking antibodies abrogate LPS-driven cytokine production. IL-6, IFNγ and IL-10 responses in response to acute activation with 0.5 ng/ml LPS plus/minus blocking antibodies are shown. Bars represent mean population responses +/− SEM.
(0.29 MB TIF)

Figure S2 LPS-driven IL-10 production is distinct between children with the homozygous (Asp/Asp+Thr/Thr) and heterozygous (Asp/Gly+Thr/Ile) TLR4 haplotypes in a sub-population that experienced infantile bronchiolitis. IL-10 responses in response to acute activation with 0.5 ng/ml LPS are shown with black bars represent median population responses, derived from individual children. * P = 0.03.
(0.33 MB TIF)
