# Supplementary figures and images for: TLR4 Asp299Gly and Thr399Ile Polymorphisms: No Impact on Human Immune Responsiveness to LPS or Respiratory Syncytial Virus
Source: PLoS One. 2010 Aug 10;5(8):e12087. doi: 10.1371/journal.pone.0012087 (PMC2919413; doi:10.1371/journal.pone.0012087)

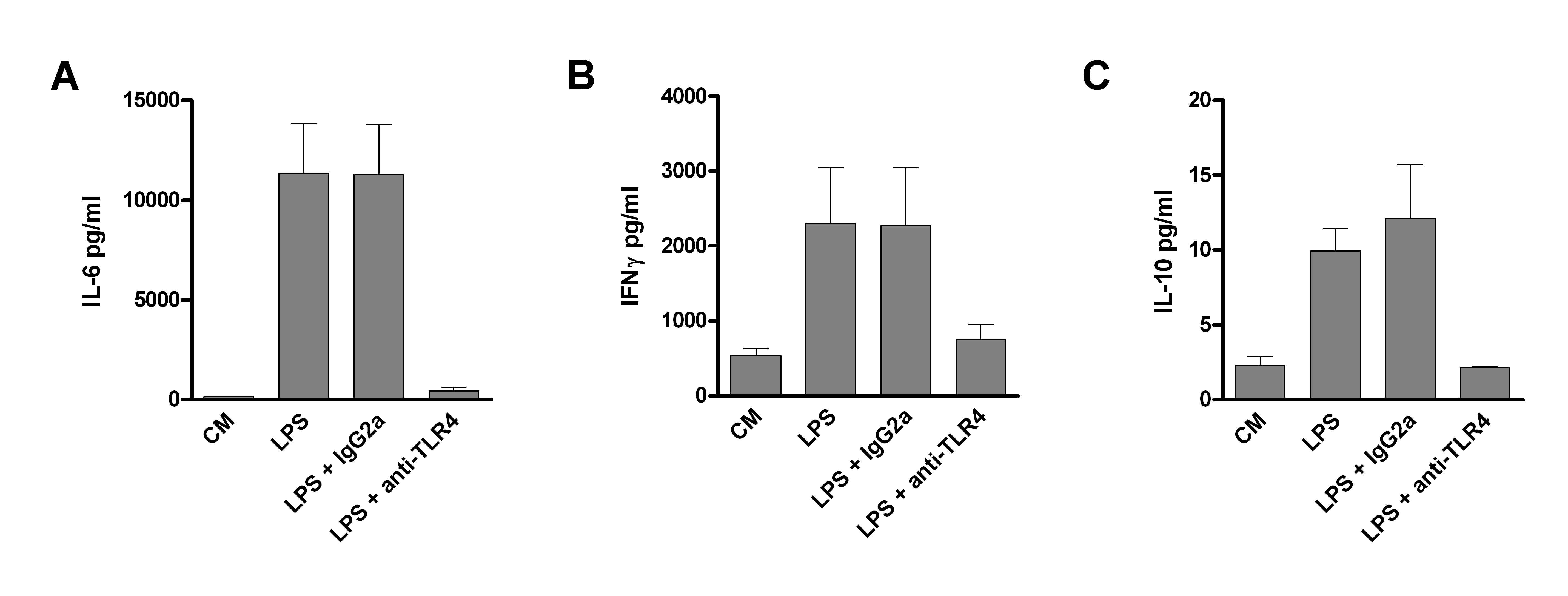

Supplement: Figure S1 — Anti-TLR4 blocking antibodies abrogate LPS-driven cytokine production. IL-6, IFNγ and IL-10 responses in response to acute activation with 0.5 ng/ml LPS plus/minus blocking antibodies are shown. Bars represent mean population responses +/− SEM. (0.29 MB TIF) [file pone.0012087.s001.tif]

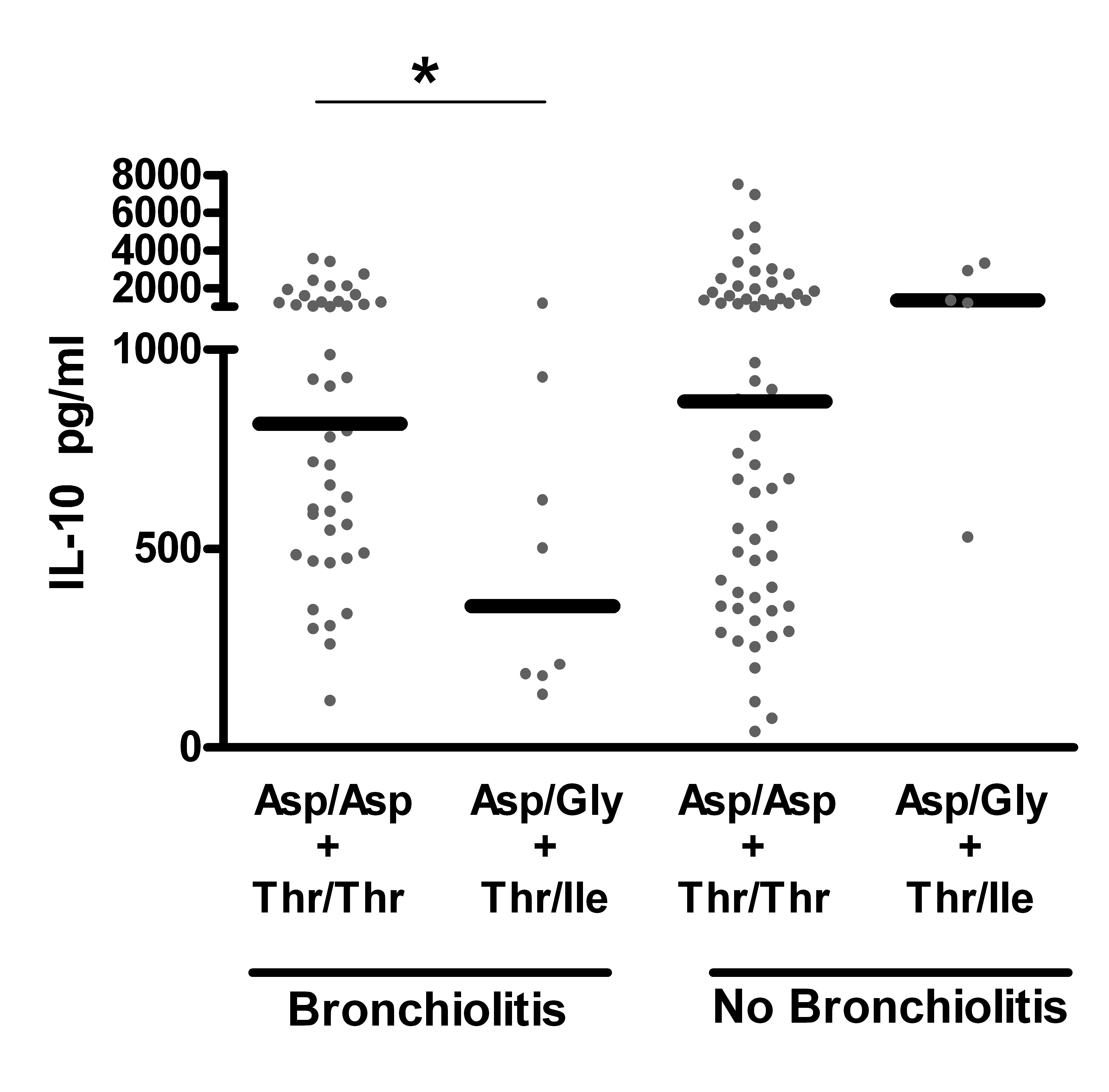

Supplement: Figure S2 — LPS-driven IL-10 production is distinct between children with the homozygous (Asp/Asp+Thr/Thr) and heterozygous (Asp/Gly+Thr/Ile) TLR4 haplotypes in a sub-population that experienced infantile bronchiolitis. IL-10 responses in response to acute activation with 0.5 ng/ml LPS are shown with black bars represent median population responses, derived from individual children. * P = 0.03. (0.33 MB TIF) [file pone.0012087.s002.tif]
